# Effects of the Synthetic Neurosteroid: 3β-Methoxypregnenolone (MAP4343) on Behavioral and Physiological Alterations Provoked by Chronic Psychosocial Stress in Tree Shrews

**DOI:** 10.1093/ijnp/pyv119

**Published:** 2015-10-17

**Authors:** Lucie Parésys, Kerstin Hoffmann, Nicolas Froger, Massimiliano Bianchi, Isabelle Villey, Etienne-Emile Baulieu, Eberhard Fuchs

**Affiliations:** MAPREG SAS, Le Kremlin-Bicêtre, France (Drs Parésys, Froger, Bianchi, Villey, and Baulieu); German Primate Center, Göttingen, Germany (Drs Hoffmann and Fuchs).

**Keywords:** Psychosocial stress, antidepressant, neurosteroids, microtubules, tree shrews

## Abstract

**Background:**

Most currently available active antidepressant drugs are selective serotonin/noradrenaline reuptake inhibitors. However, as their clinical efficacy is not immediate, long-term administration is often accompanied by substantial side effects, and numerous patients remain non- or partial responders. We have recently found that the synthetic neurosteroid derivative 3β-methoxypregnenolone, which binds to the microtubule-associated protein-2, can provide a novel therapeutic approach in experimental model of depressive disorders in rats. To further validate the antidepressant-like efficacy of 3β-methoxypregnenolone, we investigated effects of a longer treatment (4-week oral administration; 50mg/kg/d) in a nonrodent species, the tree shrew, exposed to psychosocial stress that elicits close-to-human alterations observed in patients with depressive disorders.

**Methods:**

During the experimental period, physiological parameters were registered, including core body temperature and electroencephalogram, while animals were videotaped to analyze their avoidance behavior. Morning urine samples were collected for measurements of cortisol and noradrenaline levels.

**Results:**

We found that treatment with 3β-methoxypregnenolone abolished stress-triggered avoidance behavior and prevented hormone hypersecretion, hypothermia, and sleep disturbances, further suggesting its antidepressant-like efficacy. Comparative treatment with fluoxetine also prevented some of the physiological alterations, while the hypersecretion of cortisol and sleep disturbances were not or partially restored by fluoxetine, suggesting a better efficacy of 3β-methoxypregnenolone. Alpha-tubulin isoforms were measured in hippocampi: we found that 3β-methoxypregnenolone reversed the specific decrease in acetylation of α-tubulin induced by psychosocial stress, while it did not modify the psychosocial stress-elicited reduction of tyrosinated α-tubulin.

**Conclusions:**

Taken together, these data strongly suggest a potent antidepressant-like effect of 3β-methoxypregnenolone on translational parameters.

## Introduction

Depressive disorders (DDs) are commonly observed in humans, with a lifetime prevalence of at least 10% to 15% (Kessler et al., 2003). The pathophysiology of DDs involves an alteration of the central monoaminergic system (Hamon and Blier, 2013). Hence, most currently used antidepressant drugs are selective serotonin/noradrenaline reuptake inhibitors that modulate monoaminergic neurotransmission. However, their delayed clinical efficacy, with about one-half of target patients remaining non- or partial-responders (Trivedi et al., 2006), and their adverse side effects responsible for discontinuation of treatment (Hamon and Blier, 2013) lead to a real clinical need for the development of a novel class of antidepressant molecules displaying both immediate and prolonged activities, with reduced side effects.

Based on both neuroimaging and postmortem studies in depressed subjects, it has been observed that DDs are closely associated with a reduced volume of relevant brain regions, such as hippocampi (MacQueen et al., 2003). These modifications may result from alterations of neuroplasticity (McEwen, 1999; Duman, 2002) that involve the cytoskeleton (Reines et al., 2004) and particularly the microtubular system. In line with these findings, recent studies have suggested abnormalities of the brain microtubular system in animal models of DDs (Bianchi et al., 2006; Yang et al., 2009).

Pregnenolone was found to be a modulator of the neuronal microtubular system through its binding to microtubule associated protein 2 (MAP2) (Murakami et al., 2000) and its interaction with a microtubule plus end tracking protein (CLIP170) (Weng et al., 2013). Hence, pregnenolone is able to promote microtubule assembly (Hsu et al., 2006) and neurite outgrowth (Fontaine-Lenoir et al., 2006). The synthetic compound 3β-methoxypregnenolone (MAP4343), devoid of any hormonal activity by itself or via its metabolism, was also described as a modulator of microtubular system (Fontaine-Lenoir et al., 2006). Interestingly, MAP4343 has recently displayed acute antidepressant-like activity in a validated rat model of DDs (Bianchi and Baulieu, 2012).

To further demonstrate the antidepressant-like activity of MAP4343, we used a translational model for human depressive states. The tree shrew (*Tupaia belangeri*) is a day-active animal phylogenetically close to primates, as shown by recent genome analysis (Fan et al., 2013). Prolonged psychosocial stress was created in male tree shrews by a recurrent introduction of one male into the territory of another male to develop a dominant/subordinate relationship. Importantly, the biobehavioral responses observed in subordinate tree shrews are similar to the symptoms observed in depressed patients. Psychosocial stress in tree shrews is thus considered to be a suitable model for the validation of antidepressant drugs, with a strong face validity (for review, see Fuchs, 2005).

The aim of this study was to assess the efficacy of MAP4343 over a longer period than previously described in rats (Bianchi and Baulieu, 2012), with oral drug administration, that is more reliable to future applications in patients. We found that 4-week daily administration of MAP4343 counteracted the stress-induced alterations, including social avoidance, cortisol and noradrenaline increase, elevation of core body temperature, and sleep disturbances, suggesting its potent and prolonged antidepressant-like efficacy.

## Materials and Methods

### Animal Maintenance

Adult male tree shrews of ~2 years of age from the breeding colony of the German Primate Center (Göttingen, Germany) were used. Animals were kept singly housed from puberty onwards in steel cages (50×80×125cm). Each cage contained a wooden nest box at floor level (18×15×15cm) and a system of wooden tree branches at various levels. All animals were housed under a 12-hour-light/12-hour-dark cycle (lights on at 8:00 am; 200 lux) with 60±7% relative humidity and an ambient temperature of 27±1°C (for details, see Fuchs and Corbach-Söhle, 2010). The animals had *ad libitum* access to water and food (Altromin, Lage, Germany). For this study, the minimum number of animals required to obtain consistent data was used. All animal experiments were performed in accordance with the European Communities Council Directive of September 2010 (2010/63/EU) and were approved by the Lower Saxony Federal State Office for Consumer Protection and Food Safety, Germany.

### Drug Preparation and Oral Administration

**Figure 1.**
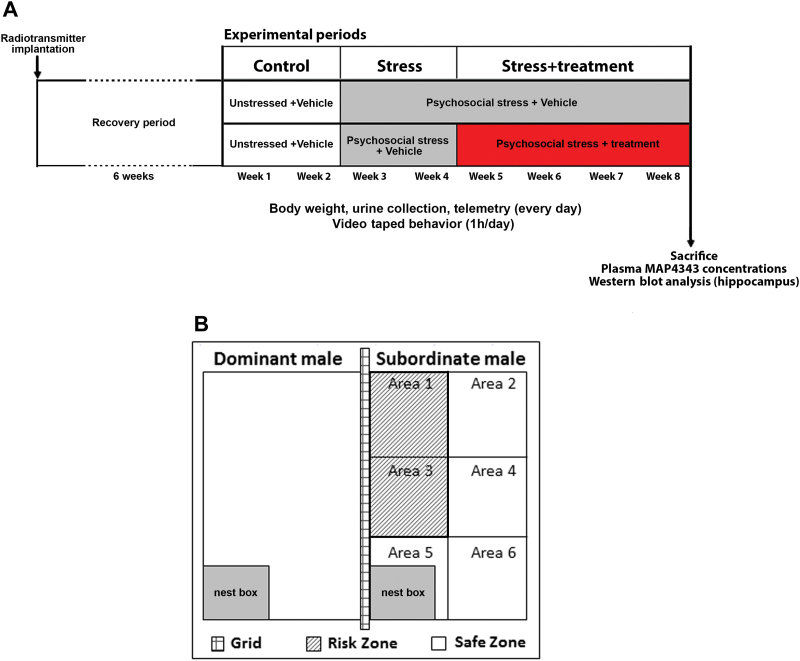
Experimental design. A: Scheme depicting the timing of psychosocial stress and oral administration of MAP4343, fluoxetine or vehicle, respectively. During the recovery period (6 weeks) following the radiotransmitter implantation, the animals remained undisturbed. The stress procedure followed the standard protocol described by Schmelting et al. (2014). B: Schematic representation of cages: the cage of a subordinate male tree shrew was separated by a grid from the cage of a dominant male, and its front panel was divided in six areas in order to measure the locomotor activity of animals. Two areas (area 1 and area 3) adjacent to the cage of the dominant male were considered as a risk zone, whereas the other areas (2, 4, 5, 6) constituted a safe zone.

MAP4343 was dissolved in 0.5% hydroxyethylcellulose by sonication during 3 cycles of 15 pulses separated by 15 seconds on ice (Branson Sonifer-450). Animals received per os administration of MAP4343 (50mg/kg/d), fluoxetine (15mg/kg/d; Fluoxetin ratiopharm Lösung, Ratiopharm, Ulm, Germany), or the vehicle (hydroxyethylcellulose) each day during the treatment period (Figure 1A). Drugs were administrated between 8:00 and 8:30 am. A detailed methodology for oral pharmacological treatment in tree shrews was described by Schmelting et al., 2014.

### Psychosocial Stress in Tree Shrews

As depicted in Figure 1A, our experimental procedure of psychosocial stress involved 3 separated groups composed by tree shrews treated with either MAP4343 or fluoxetine, and untreated animals receiving vehicle alone (n=8 for each group).

During an initial 2-week control period (Wks 1&2), animals were separately housed in an unstressed condition with a daily administration of vehicle. In the next 6-week period, psychosocial stress was applied to tree shrews. It consisted of the introduction of a naïve animal into the cage (“territory”) of a socially experienced male. After this first direct contact, the 2 males stayed in auditory, olfactory, and visual proximity in their respective cages separated with the wire grid (Figure 1B). Once per day, the grid was removed for approximately 1 hour between 8:00 am and 11 am, allowing interaction between the 2 animals. The animals engaged in a competition over the “territory” and established a dominant/subordinate relationship. To exclude the effects of individual differences in the intensity of attacks by the dominant male and to avoid habituation, the subordinate animal was confronted daily with another dominant male according to a Latin square design. During the first 2 weeks of stress (Wks 3–4), tree shrews received vehicle. In the last 4 weeks of stress (Wks 5–8), MAP4343 or fluoxetine was daily administered to the treated groups while the untreated group received only vehicle (Figure 1). During the whole experimental period, animals were videotaped for 1 hour (4:30 pm to 5:30 pm) each day in order to evaluate behavioral parameters, while telemetry was continuously operated. In addition, body weight was measured and morning urine was collected every day before drug administration. At the end of the procedure, animals were sacrificed by decapitation. Blood was collected to measure plasma concentrations of MAP4343, and hippocampi were isolated, immediately frozen, and stored at -80°C until analyses.

### Behavioral Experiments

Behavioral experiments consisted of measurement of locomotor activity (LMA) and avoidance behavior. For this purpose, the front panels of the cages were divided in 6 approximately equal areas (Figure 1B). All animals were videotaped directly in their cages each day for 1 hour in the late afternoon (from 4:30 pm to 5:30 pm). Data were then analyzed using EthoVision Pro 3.1 software (Noldus, Wageningen, The Netherlands).

#### Measurement of Locomotor Activity (LMA)

LMA was recorded as an

individual′s frequency of crossings between adjacent areas in the cage (Kramer et al., 1999). The individual effect of psychosocial stress and drug-induced changes in LMA were evaluated by counting the number of border crossings between the defined areas.

#### Evaluation of Avoidance Behavior

Areas 1 and 3, adjacent to the dominant animal’s cage,

were defined as a risk zone, while the zone constituted by areas 2, 4, 5, and 6 represented a safe zone (Figure 1B). Hence, the evaluation of avoidance behavior consisted of the measurement of 2 parameters: the number of border crossings between areas 1 and 3 and the total time (in minutes) spent in areas 1 and 3.

### Measurements of Urinary Hormones

Urinary samples were collected each morning before the lights were turned on, under dimmed light, as previously described by Schmelting et al. (2014) (see supplementary Material for more details).

### Core Body Temperature

CBT was recorded once per minute throughout the 24-hour period (except for the 2 hours between 8 am and 10 am) via a telemetric radiotransmitter (PhysioTel F40-EET; DSI, St. Paul, MN; receiver plates were RMC-1 from DSI) surgically implanted in the peritoneal cavity (Coolen et al., 2012). The lowest value, the nadir, was specifically studied.

### Sleep Recordings

Electroencephalogram (EEG) recordings were performed, using the same telemetric devise (PhysioTel F40-EET), from 6 pm to 8 am according the procedure described in detail by Coolen et al. (2012). EEG analyses facilitated the distinction between waking, rapid eye movement sleep (REM), and non-rapid eye movement sleep (nREM). Total sleep time (in minutes) was calculated by the addition of REM sleep phases and nREM sleep phases. Because short wakefulness phases occur during sleep in tree shrews (Coolen et al, 2012), the measurement of total sleep time did not include these wakefulness phases. The sleep period is defined as the period between the first and last sleep episode during the dark phase (including the short wakefulness phases), and sleep efficiency was determined as the ratio between the total sleep time and the duration of the sleep period.

### Determination of MAP4343 Concentration in Plasma

MAP4343 concentrations were measured by liquid chromatography/tandem mass spectrometry following a method developed for Mapreg by Bertin Pharma (Orleans, France). A full description of the methodology is provided in the supplementary Material.

### Western-Blot Detection of Hippocampal Tubulin Isoforms

Infrared Western-blot analyses were performed on hippocampus extracts in stressed tree shrews treated by MAP4343 or untreated. Data were compared with those obtained in a group of unstressed animals (~2 years old) that received vehicle solution daily and at the same time as treatments given to stressed groups. Total α-tubulin (Tot-Tub) tyrosinated α-tubulin (Tyr-tub), detyrosinated α-tubulin (Glu-tub), and acetylated α-tubulin (Acet-tub) were quantified by using the Odyssey imaging system, as fully described in the supplementary Material.

### Statistical Analyses

Data were daily collected and weekly means (±SEM) were calculated. Statistical analyses, performed using InVivoStat Statistical Software, are detailed in the supplementary Material.

## Results

### Plasma MAP4343 Concentrations

We found that all treated tree shrews displayed measurable plasma levels of MAP4343 in a nanomolar range; the mean concentration after the 4-week administration reached 118.7±32.3nM. Variable MAP4343 plasma concentrations are obviously not surprising if we consider that our compound was orally administrated.

### Behavioral Parameters

#### Locomotor Activity (LMA)

**Figure 2.**
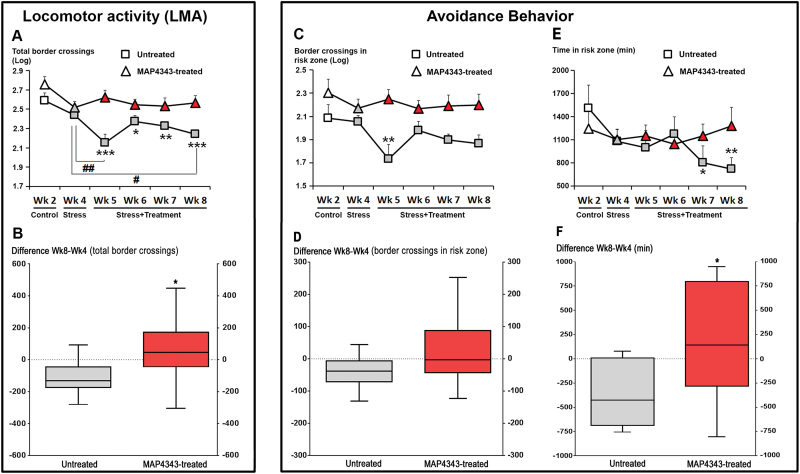
Effects of 4-week administration of MAP4343 on LMA and avoidance behavior in stressed tree shrews. A: Curves representing total LMA recorded during the whole experimental period in animals receiving vehicle (squares) or treated with MAP4343 (triangles). Data, expressed in log space, are weekly means±SEM calculated from daily measurements in independent animals (n=8 per group). B: Boxplots showing changes of total LMA (total border crossings) between the start and the end of the stress period in untreated (grey bar) or MAP4343-treated group. Data are difference scores obtained between Wk8 and Wk4. *P<.05 (unpaired Student’s t test). C, E: Curves representing the numbers of border crossings (C) and the time spent (E) in the risk zone during the whole experimental period in animals receiving vehicle (squares) or treated with MAP4343 (triangles). Data, expressed in log space (C) or in minutes (E), are weekly means±SEM calculated from daily measurements in independent animals (n=8 per group). D, F: Boxplots showing changes of border crossings (D) or of time spent (F) in risk zone between the start and the end of the stress period in untreated (grey bar) or MAP4343-treated (red bar) group. Data are difference scores obtained between Wk8 and Wk4. *P<.05 (unpaired Student’s t test). In A, C, E: white items represent the values obtained during the control period, grey items represent the values obtained during the stress period without treatment and red items represent the values obtained during the stress period with MAP4343 treatment. *P<.05, **P<.01 and ***P<.001 when compared to the control period (Wk2); ^#^ *P<.*05 and ^##^ *P<.*01 when compared between the indicated weeks (One-way ANOVA for repeated measures, followed by a Fisher’s LSD test to compare weekly means within each respective group).

In animals receiving vehicle, psychosocial stress affected LMA (F_5,35_=6.05, *P*<.001; 1-way ANOVA with repeated measures): a significant decrease in activity was found during most weeks of the stress period when compared with the control period (Wk2; Figure 2A, squares). In addition, LMA displayed a time-dependent reduction within the stress period; the value measured at the end of the stress period (Wk8) was significantly reduced compared with the first stress period (Wk4, *P<.*05). When animals received a 4-week oral administration of MAP4343, psychosocial stress did not significantly modify LMA (F_5,35_=1.89, *P*=0.12; 1-way ANOVA with repeated measures; Fig. 2A, triangles). Thus, MAP4343 prevented a decrease of LMA during the social stress exposure. The difference score of total LMA between the end and the beginning of the stress period (Wk8 vs Wk4) was found negative in tree shrews receiving vehicle (-108.7±39.1, mean±SEM), whereas it was positive (+58.5±76.0) and significantly different (*P<.*05 compared to untreated group, Student’s t test) in MAP4343-treated animals (Fig. 2B).

#### Avoidance Behavior

In tree shrews receiving vehicle, psychosocial stress was found to significantly reduce the number of border crossings in the risk zone (F_5,35_=2.65; *P<.*05, one-way ANOVA with repeated measures; Fig. 2C, squares), suggesting that stress triggered an avoidance behavior, as previously described by Kramer et al. (1999). By contrast, when tree shrews were treated with MAP4343, no significant change in the number of border crossings in this zone was observed during social stress (F_5,35_=0.62; p=0.69, one-way ANOVA with repeated measures; Fig. 2C, triangles). The difference score of number of border crossings in risk zone between Wk8 and Wk4 was found negative in stressed tree shrews receiving vehicle (-38.7±18.5, mean±SEM), whereas it appeared positive in MAP4343-treated animals (+22.3±40.2, mean±SEM), although these values were not statistically different (p=0.09, Student’s t test; Fig. 2D).

Furthermore, we investigated the time spent in both areas 1 and 3, defining a second parameter for the assessment of avoidance behavior. Tree shrews receiving vehicle showed a progressive decrease in time spent in the risk zone during the stress period (F_5,35_=2.24, p=0.07, one-way ANOVA with repeated measures; Fig. 2E, squares). The values measured during the two last weeks of the stress period (Wk7 and Wk8) were significantly lower than those measured during the control period (Wk2). In contrast, 4-week treatment with MAP4343 prevented the decrease in time spent in the risk zone during the stress period (F_5,35_=0.36, p=0.87, one-way ANOVA with repeated measures; Fig. 2E, triangles). The difference score of the time spent in the risk zone between Wk8 and Wk4 was strongly negative in stressed tree shrews receiving vehicle (-361.3±115.9; mean±SEM), whereas this score was found positive in MAP4343-treated animals (+173.3±220.4, mean±SEM; Fig. 2F) and significantly different as compared to untreated group (*P<.*05, Student’s t test)

Taken together these data strongly suggest that continuous administration of MAP4343 can prevent the psychosocial stress-triggered avoidance behavior in tree shrews.

### Neuroendocrine Function: Urinary Hormones

#### Cortisol Concentrations

**Figure 3.**
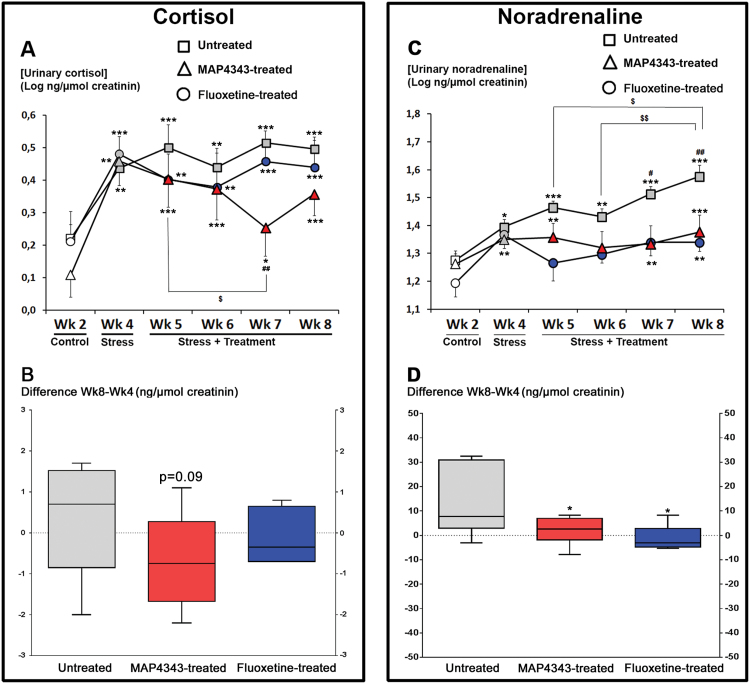
Effects of 4-week administration of MAP4343 and fluoxetine on urinary hormone levels in stressed tree shrews. A, C: Curves representing urinary cortisol (A) or noradrenaline (C) concentrations during the whole experimental period in animals receiving vehicle (squares), MAP4343 (triangles) or fluoxetine (circles). Data, expressed as the log of ng of cortisol or noradrenaline per µmol of creatinine, are weekly means±SEM calculated from daily measurements in independent animals (n=5–8). White items represent the values obtained during the control period, grey items represent the values obtained during the stress period without treatment and red or blue items represent the values obtained during the stress period with MAP4343 or fluoxetine treatment, respectively. *P<.05, **P<.01 and ***P<.001 when compared to the control period (Wk2); ^#^ *P<.*05 and ^##^ *P<.*01 when compared to the first stress period (Wk4); ^$^ *P<.*05 and ^$$^ *P<.*01 when compared between the indicated weeks (One-way ANOVA for repeated measures followed by a Fisher’s LSD test to compare weekly means within each respective group). B, D: Boxplots showing changes of urinary cortisol (B) or noradrenaline (D) concentrations between the beginning and the end of the stress period in untreated (grey bar), MAP4343-treated (red bar) or fluoxetine-treated (blue bar) group. Data are difference scores obtained between Wk8 and Wk4. *P<.05 and **P<.001 as compared to untreated group (One-way ANOVA followed by a Fisher’s LSD post hoc test).

The psychosocial stress in tree shrews only receiving vehicle strongly increased cortisol concentrations (F_5,35_=6.40, *P<.*001, one-way ANOVA with repeated measures, n=8; Fig. 3A, squares). This increase was measured during the beginning of the stress period (Wk4) and then remained stable until the end of the period (Wk8). In tree shrews treated with MAP4343, psychosocial stress also triggered a cortisol increase (F_5,35_=7.29, *P<.*001, one-way ANOVA with repeated measures, n=8; Fig. 3A, triangles), but the 4-week treatment was able to significantly reduce such cortisol increase during the third week of treatment (Wk7) when compared to Wk4 and Wk5, respectively. In the fluoxetine-treated animals, the psychosocial stress induced a significant increase of urinary cortisol concentrations (F_5,25_=5.91, *P<.*001, one-way ANOVA with repeated measures, n=6; Fig. 3A, circles). Fluoxetine treatment did never significantly modify the increased cortisol concentrations at any point during the stress period.

The difference scores between Wk8 and Wk4 were not found significantly different between groups (F_2,19_=1.52, p=0.24, one-way ANOVA). The score was positive in stressed tree shrews receiving vehicle (+0.3±0.4, mean±SEM; Fig. 3B), while it was negative in MAP4343-treated animals (-0.7±0.4, mean±SEM), but not significant when compared to the untreated group (p=0.09, Fisher’s LSD test; Fig. 3B). In fluoxetine-treated animals, the difference score was found slightly negative (-0.1±0.2, mean±SEM) and not significant when compared to untreated group (p=0.46, Fisher’s LSD test, Fig. 3B).

#### Noradrenaline Concentrations

In animals receiving vehicle, the psychosocial stress triggered a hypersecretion of urinary noradrenaline (F_5,30_=8.75, *P<.*001, one-way ANOVA with repeated measures, n=7; Fig. 3C, squares). Thus, the noradrenaline concentration was higher during the first stress period (Wk4) as compared to the control period (Wk2), and these significant increases grew progressively and significantly during the stress period. In tree shrews treated with MAP4343, an increase in noradrenaline was also observed during the stress period (F_5,30_=3.30, *P<.*05, one-way ANOVA with repeated measures, n=7; Fig 3C, triangles), but the treatment resulted in the stabilization of the stress-induced noradrenaline increase. Psychosocial stress also triggered a significant increase of urinary noradrenaline concentration in fluoxetine-treated animals (F_5,20_=3.21, *P<.*05, one-way ANOVA with repeated measures, n=5; Fig. 3C, circles) and fluoxetine treatment abolished the progression of the noradrenaline increase during the stress period.

The difference scores between Wk8 and Wk 4 were found significantly different between the three groups (F_2,16_=4.72, *P<.*05, one-way ANOVA). They were reduced both in MAP4343-treated (1.9±2.2, mean±SEM) and fluoxetine-treated (-1.9±1.6, mean±SEM) animals when compared to the stressed tree shrews receiving vehicle alone (13.7±5.2, mean±SEM; *P<.*05, Fisher’s LSD test; Fig. 3D)

### Core Body Temperature

#### Temperature Area Under Curve (AUC)

**Figure 4.**
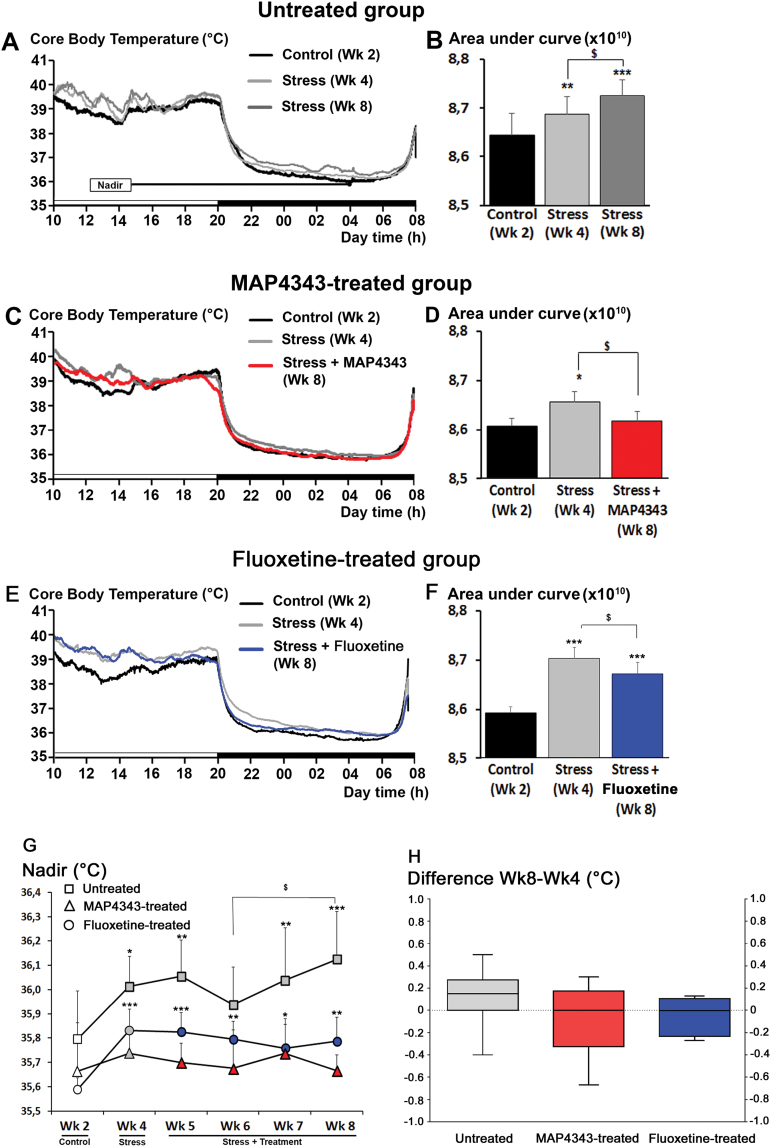
Effects of 4-week administration of MAP4343 and fluoxetine on core body temperature (CBT) in stressed tree shrews. A, C, E: Representative curves showing the CBT recorded during the unstressed control period (Wk2; black lines), the first stress period (Wk4, light grey lines) and the last stress period (Wk8, dark grey, red or blue lines) in animals receiving vehicle (A), MAP4343 (C) or fluoxetine (E). The black-and-white bar at the bottom of the graphs indicates the 12-hr dark/12-hr light cycle (light from 08:00 a.m to 8:00 p.m.). B, D, F: Histograms representing the respective areas under curves measured during Wk2 (dark bars), Wk4 (light grey bars) and Wk8 (dark grey, red or blue bars) in animals receiving vehicle (B), MAP4343 (D) or fluoxetine (F), respectively (n=8 independent animals per group). *P<.05, **P<.01 when compared to the control period (Wk2); ^$^ *P<.*05 when compared to the first stress period (Wk4) (One-way ANOVA for repeated measures followed by a Fisher’s LSD test to compare weekly means within each respective group). G: Curves representing the nadir of CBT measured during the whole experimental period in animals receiving vehicle (squares), MAP4343 (triangles), or fluoxetine (circles). White items represent the values obtained during the control period, grey items represent the values obtained during the stress period without treatment and red or blue items represent the values obtained during the stressed period with MAP4343 or fluoxetine treatment, respectively. Data are weekly means±SEM calculated from daily measurements in each independent animal (n=8 per group). *P<.05, **P<.01 and ***P<.001 when compared to the control period (Wk2); ^$^ *P<.*05 when compared between the indicated weeks (One-way ANOVA for repeated measures followed by a Fisher’s LSD test to compare means from each week within each respective group). H: Boxplots showing changes of nadir of CBT between the beginning and the end of the stress period in untreated (grey bar), MAP4343-treated (red bar) or fluoxetine-treated (blue bar) group. Data are difference scores obtained between Wk8 and Wk 4. *P<.05 and **P<.001 as compared to untreated group (One-way ANOVA followed by a Fisher’s LSD post hoc test).

In animals receiving vehicle, exposure to psychosocial stress induced an increase in core body temperature (CBT; F_2,14_=15.4, *P<.*001, one-way ANOVA with repeated measures, n=8; Fig. 4A) during both diurnal and nocturnal phases. The resulting AUC for total temperature (from both diurnal and nocturnal phases) was significantly increased during the beginning stress period (Wk4) when compared to the control period (Wk2). Moreover, this hyperthermia was further increased at the end of the stress period (Wk8) as compared to the first stress period (Wk4; Fig. 4B). When animals were treated with MAP4343, the stress-induced hyperthermia observed during the first stress period (Wk4) was significantly reduced and almost abolished during the last stress period (Wk8) (F_2,14_=4.89; *P<.*05, one-way ANOVA with repeated measures, n=8; Fig. 4C,D), since the CBT value became not significant when compared to the one from the control period (Wk2; Fig. 4D). Measurements of CBT in fluoxetine-treated animals revealed that psychosocial stress triggered hyperthermia (F_2,14_=46.11, *P<.*001, one-way ANOVA with repeated measures, n=8; Fig. 4E). Fluoxetine administration resulted in a significant reduction of stress-induced hyperthermia at the end of the stress period (Wk8; Fig. 4F). However, the CBT value from Wk8 remained significantly higher than the one from the control period (Wk2, Fig. 2F).

#### Nadir for the Nocturnal Core Body Temperature

Nadir values from nocturnal CBT were significantly increased during stress period in animals receiving vehicle (F_5,35_=3.40, *P<.*05, one-way ANOVA with repeated measures, n=8; Fig. 4G, squares). This increase was found significant during the first stress period (Wk4) and potentiated with the continuation of the stress period, since the value observed during the last stress period (Wk8) was significantly increased when compared to the one from the third stress period (Wk6). By contrast, when tree shrews were treated with MAP4343, the nadir values were not significantly modified during the stress period (F_5,35_=0.15, p=0.97, one-way ANOVA with repeated measures, n=8; Fig. 4G, triangles). The nadir of nocturnal CBT appeared not significantly increased at first stress period (Wk4), contrary to that observed in the untreated group, suggesting some variability of the stress action on CBT during the first stress period. The progression of nadir until the end of the stress period was abolished in MAP4343-treated group. In fluoxetine-treated animals, stress induced a significant increase of nadir values of nocturnal CBT (F_5,35_=4.13, *P<.*001, one-way ANOVA with repeated measures, n=8; Fig. 4G, circles). However, the nadir increase observed during the first stress period was not potentiated at any other point of the stress period.

Measurements of difference scores of nadir revealed no significant differences between three groups (F_2,21_=1.18, p=0.32, one-way ANOVA), despite of lower values observed in MAP4343-treated (-0.07±0.11, mean±SEM) and fluoxetine-treated (-0.04±0.05, mean±SEM) animals as compared to stressed tree shrews receiving vehicle (0.11±0.09, mean±SEM; Fig. 4H).

### Sleep Patterns

#### Total Sleep Time

**Figure 5.**
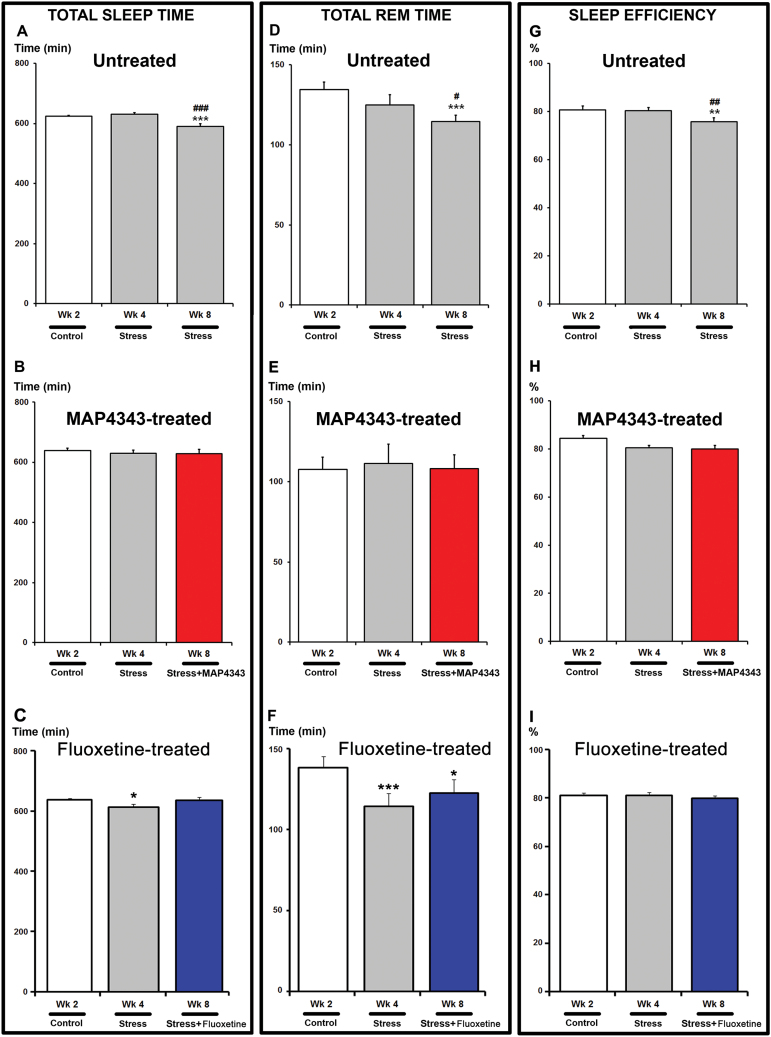
Effects of 4-week administration of MAP4343 and fluoxetine on total sleep, REM and sleep efficiency in stressed tree shrews. Histograms representing total sleep time (A-C), total REM sleep time (D-F) and sleep efficiency (G-I), evaluated by EEG recordings during the whole experimental period in animals receiving vehicle (A, D, G), MAP4343 (B, E, H) or fluoxetine (C, F, I). Data, expressed as minutes (A-F) or as percentage of sleep efficiency (G-I), are means±SEM from independent animals (n=7 for each group). White bars represent the values obtained during the control period, grey bars represent the values obtained during the stress period without treatment and red or blue bars represent the values obtained during the stressed periods with MAP4343 or fluoxetine treatment, respectively. *P<.05, **P<.01 and ***P<.001 when compared to the control period (Wk2); ^#^ *P<.*05, ^##^ *P<.*05 and ^###^ *P<.*05 when compared to the first stress period (Wk4) (One-way ANOVA for repeated measures followed by a Fisher’s LSD post-hoc test to compare weekly means within each respective group).

Reduction of the total sleep time was observed in stressed tree shrews receiving vehicle (F_5,30_= 6.47, *P<.*001, one-way ANOVA with repeated measures, n=7; Fig. 5A). Indeed, a significant decrease was reached during the last week of the stress period (Wk8) when compared to that measured during the control period (Wk2) and the first stress period (Wk4). Upon treatment with MAP4343 for 4 weeks, the alteration in total sleep time induced by the social stress was abolished (F_5,30_=0.42, p=0.83 one-way ANOVA with repeated measures, n=7, Fig. 5B). In fluoxetine-treated animals, total sleep time was found significantly reduced by the psychosocial stress (F_5,30_=2.69, *P<.*05, one-way ANOVA with repeated measures, n=7). Interestingly, the significant decrease observed during the first stress period (Wk4) has disappeared during the last stress period (Wk8) after the 4-week fluoxetine administration (Fig. 5C).

#### REM Sleep

The REM time was significantly reduced by psychosocial stress in animals receiving vehicle (F_5,30_=4.22, *P<.*05, one-way ANOVA with repeated measures, n=7; Fig. 5D). The values were found to be lower during the final stress week (Wk8) when compared to the control period (Wk2) and to the first stress period (Wk4). The 4-week MAP4343 administration prevented the stress-induced alteration of total REM sleep time (F_5,30_=0.30, p=0.91, one-way ANOVA with repeated measures, n=7; Fig. 5E). In fluoxetine-treated animals, the REM sleep time was significantly altered (F_5,30_=4.87, *P<.*01, one-way ANOVA with repeated measures, n=7; Fig. 5F) during the stress period. A significant decrease was found during the first stress period (Wk4) and the fluoxetine treatment failed to reverse such reduction of REM sleep time during the last stress period (Wk8; Fig. 5F).

#### Sleep Efficiency

In animals receiving only vehicle solution, psychosocial stress induced a significant alteration of sleep efficiency (F_5,30_=3.35, p=0.016, one-way ANOVA with repeated measures, n=7; Fig. 5G). In MAP4343-treated group, the decrease in sleep efficiency was not significant (F_5,30_=1.92, p=0.12, one-way ANOVA with repeated measures, n=7; Fig. 5G) suggesting that the 4-week administration of MAP4343 prevented the alteration of this parameter. Similarly, stressed tree shrews treated with fluoxetine did not display any significant alteration of sleep efficiency during the stress period (F_5,30_=0.49, p=0.78, one-way ANOVA, n=7; Fig. 5H).

### Western Blot Quantification of Hippocampal α-Tubulin Isoforms

**Figure 6.**
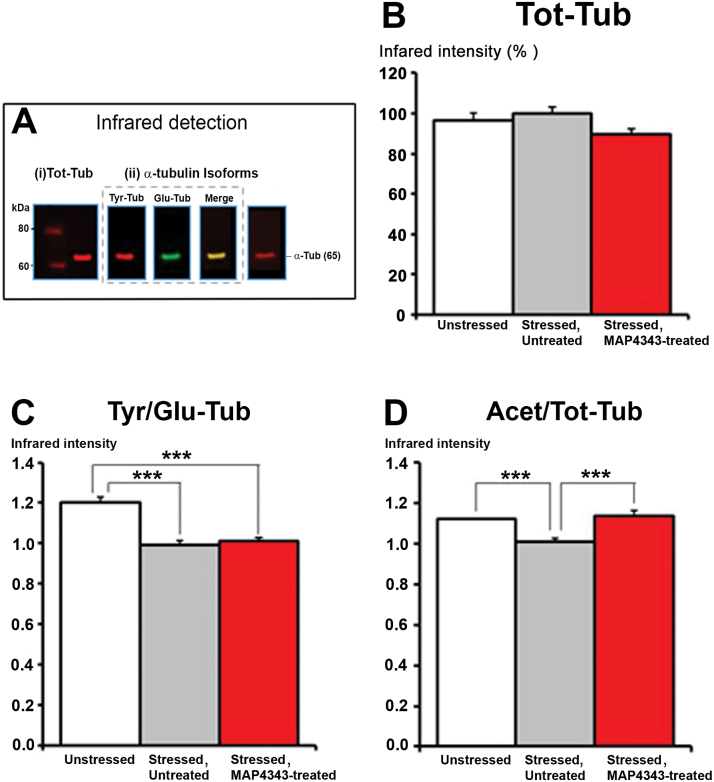
Effects of 4-week administration of MAP4343 on alpha-tubulin isoforms in hippocampi from stressed tree shrews. A: Representative Western blot showing infrared specific detection of proteins: (i) total α-Tubulin in red (Tot-Tub), (ii) α-tubulin isoforms, i.e. tyrosinated α-Tubulin in red (Tyr-Tub), detyrosinated α-Tubulin in green (Glu-Tub) and acetylated α-Tubulin in red (Acet-Tub). B-D: Histograms showing the Tot-Tub (B), the Tyr/Glu-Tub ratio (C), the Acet/Tot-Tub ratio (D) in control tree shrews (white bar; “Unstressed” group), in stressed tree shrews receiving vehicle (grey bar; “Stressed, Untreated” group) or in stressed tree shrews treated with MAP4343 (red bar; “Stressed MAP4343-treated” group). Data, expressed as percentage of infrared intensity (B) or ratio of two intensities (C, D) are means ± SEM obtained from independent animals (n=6 per group). ***P<.001 when compared between the indicated groups (Kruskall-Wallis ANOVA, followed by Dunn’s post-hoc test).

Quantitation of total α-tubulin (Tot-Tub), tyrosinated α-tubulin (Tyr-Tub) detyrosinated α-tubulin (Glu-Tub) and acetylated α-tubulin (Acet-Tub) were performed by infrared Western blot analyses (Fig. 6A). The Tyr/Glu tub ratio were calculated as a believed index of microtubular dynamics (Bianchi et al., 2009). Expression of Tot-tub from hippocampus was not found to be significantly different between the three groups (i.e. “Unstressed”; “Stressed Untreated” and “Stressed MAP4343-treated”; Fig. 6B). Exposure to psychosocial stress in animals receiving vehicle induced a significant reduction of the Tyr/Glu-Tub ratio, and the 4-week oral administration of MAP4343 did not significantly change the reduction of Tyr/Glu-Tub ratio induced by stress (Fig. 6C). On the other hand, social stress exposure induced a significant decrease of the Acet-Tub expression in tree shrews receiving vehicle. Interestingly, when stressed animals were treated with MAP4343, such treatment significantly restored the level of Acet-Tub expression (Fig. 6D).

## Discussion

In this study, we have demonstrated that MAP4343 exerts an antidepressant-like efficacy in tree shrews subjected to psychosocial stress, a close-to-primate model of DDs. The face validity of such a model was provided by biobehavioral changes produced by long-term stress exposure, and considered similar to symptoms observed in depressed patients (Fuchs and Flugge, 2002; Fuchs, 2005). Indeed, and as already described, we found that social stress triggered (i) a decrease of body weight, (ii) a reduced locomotion which may be related to a psychomotor retardation in patients, (iii) a cortisol hypersecretion, as described in depressed patients (Carroll et al., 2012) and (iv) an elevation of the nocturnal core body temperature as observed in patients during the depressive phase (Souetre et al., 1988). Moreover, (v) we revealed that psychosocial stress in tree shrews triggered sleep disturbances, considered as a cardinal symptom of human DDs (Adrien, 2002; Nutt et al., 2008); a finding that further reinforces the face validity of the model. The stressed tree shrews were also shown to display neurostructural changes, such as a reduced volume of hippocampus (Czeh et al., 2001) or a dendritic shrinkage (Magarinos et al., 1996), similarly to those described in depressed patients (Duman, 2002). Anhedonia, one core symptom of DDs was eventually recently evidenced in stressed tree shrews which displayed a reduced sucrose preference (Wang et al., 2013), as described in rats subjected to chronic mild stress, one of the most extensively validated animal model of depression (Willner, 2005). Importantly, the predictive validity of the tree shrew model was pinpointed by the observed treatment efficacy of both classical (fluoxetine or clomipramine) and atypical (tianeptine, NK1 antagonists, agomelatine) antidepressant drugs (Lucassen et al., 2004; van der Hart et al., 2005; Michael-Titus et al., 2008; Schmelting et al., 2014).

### MAP4343 Antidepressant-Like Efficacy

Interestingly, prolonged administration (4 weeks) of MAP4343 allowed substantial improvement of most depressive-like alterations elicited by psychosocial stress, with the exception of reduction of body weight (see Fig. S1, Supplementary Material). However, MAP4343 treatment did not worsen the stress-induced body weight loss, evoking, as observed with fluoxetine, an absence of side-effects of our compound on food intake and/or metabolism (see Fig. S1, and Table 1, Supplementary Material). Similar results were observed previously with tianeptine (Lucassen et al., 2004) and clomipramine (Wang et al., 2013) treatments,, neither of which being able to rescue the body weight decrease.

Hence, MAP4343 has successfully prevented behavioral changes such as decreased LMA and augmented avoidance behavior. The decrease of LMA appeared at the beginning of the stress period: MAP4343 readily counteracts this behavioral alteration as early as in the first week of administration and this effect persisted until the end of the stress period. Similar effects were observed with clomipramine (Fuchs et al., 1996; Wang et al., 2013), agomelatine (Schmelting et al., 2014) and the NK1 receptor antagonist, L-760735 (van der Hart et al., 2005). Social avoidance, triggered during the last two weeks of social stress, was abolished by MAP4343, suggesting a more prolonged efficacy. Such avoidance response may be associated with anxious-like behavior and the efficacy of MAP4343 to reduce this behavior may reveal its anxiolytic-like property, as previously described in rats (Bianchi and Baulieu, 2012). However, diazepam did not show any beneficial effects on stress-induced behavioral changes in tree shrews (van Kampen et al., 2000), suggesting that social avoidance might rather reflect the “depressed mood” than anxiety in this stress model.

MAP4343 displayed efficacy in reducing the increase of the adrenal cortical hormone levels triggered by social stress. Responses to stressful events involve the stimulation of the hypothalamic-pituitary-adrenal axis, leading to an increase in cortisol secretion which has been related to the pathophysiology of DDs (Holsboer, 2001). Such cortisol response, previously observed in our model (Johren et al., 1994; Meyer et al., 2001; Kozicz et al., 2008), was significantly decreased by MAP4343 administration after 3 weeks of treatment. However, this effect did not persist during the 4^th^ week of treatment, suggesting a variable efficacy of MAP4343 of cortisol on the morning urine. Differently, we never found here any efficacy of fluoxetine in reducing the stress-elicited cortisol increase (see Table 1). By contrast, Schmelting and colleagues observed that fluoxetine and agomelatine counteracted the increase of urinary cortisol concentration during the 4^th^ week of administration. An older study has shown efficacy of clomipramine to reduce cortisol concentrations in stressed tree shrews (Kramer et al., 1999). However, such effect was not systematically reproducible with this tricyclic antidepressant drug (van der Hart et al., 2005). Furthermore, MAP4343 counteracted the activation of the neurosympathetic system by blocking the time-dependent increase of noradrenaline secretion triggered by stress exposure. A similar effect was observed with fluoxetine in the present study (Table 1) and in a previous report (Schmelting et al., 2014).

**Table 1.** Efficacy of MAP4343 vs Fluoxetine to Prevent Changes Induced by Psychosocial Stress in Tree Shrews

	**MAP4343-Treated Group**	**Fluoxetine-Treated Group**
Body weight loss	-	-
LMA reduction	+	n.d.
Cortisol increase	+/-	-
Noradrenaline increase	+	+
CBT elevation	+	+
Sleep disturbances	+	+/-

Abbreviations: CBT, core body temperature; LMA, locomotor activity; n.d., not determined (accidental troubles in the animal room have precluded obtaining of quantitative results); + refers to significant efficacy preventing physiological changes; +/- refers to variable efficacy preventing physiological changes; - refers to an absence of efficacy preventing physiological changes;

Stress-induced hyperthermia is a well-known response to stressors in rodents (Bouwknecht et al., 2007) and in humans (Marazziti et al., 1992). In depressive patients, a clear-cut increase of nocturnal body temperature was described during the depressive phase and corrected during the remission (Souetre et al., 1988). We show that chronic social stress elicited an elevation of the CBT, as previously described (Kohlhause et al., 2011), and that MAP4343 was able to counteract this increase, similarly to agomelatine and more efficiently than fluoxetine (Schmelting et al., 2014), although in the present study fluoxetine displayed similar efficacy to MAP4343 (Table 1).

Investigations on the pathophysiology of DDs have yielded a number of highly replicable sleep abnormalities in humans (Arfken et al., 2014), mainly characterized by insomnia and fragmented sleep (Nutt et al., 2008). In tree shrews, we show for the first time that psychosocial stress leads to sleep alterations consisting in the decrease of total sleep time and the sleep efficiency observed at the end of the stress period. MAP4343 was able to normalize all these sleep alterations which are late responses to psychosocial stress. Administration of fluoxetine prevented alterations of two sleep parameters, i.e. total sleep time and sleep efficiency, but failed to prevent alteration of REM sleep time, showing a variable efficacy and suggesting a more robust efficacy of MAP4343 than fluoxetine on sleep disturbances (Table 1).

### MAP4343 and Tubulin Isoforms

Since MAP4343 may modulate the microtubular system though the binding at MAP2 (Fontaine-Lenoir et al., 2006), we assessed the expression of various isoforms of α-tubulin, as markers of microtubular dynamics. We found here that stress-induced alterations were accompanied by a specific reduction of (i) the ratio tyrosinated/detyrosinated α-tubulin and (ii) the acetylated α-tubulin in the hippocampus. Tyrosinated α-tubulin is associated with neurite regrowth (Marcos et al., 2009) and has been found to be more abundant in neo-polymerized microtubules (Peris et al., 2006), whereas detyrosinated α-tubulin is detected in polymerized tubulin over time. Alterations of tyrosinated/detyrosinated cycle may result in a reduced microtubular dynamics and suggest impairment of neuronal structures (Bianchi et al., 2009). A reduction of the ratio tyrosinated/detyrosinated α-tubulin was previously described in the hippocampus from rats subjected to various stressors (Bianchi et al., 2006; Yang et al., 2009). We found here that social stress also reduced the expression of acetylated α-tubulin, similarly to that observed after 8-week isolation in Lister Hodeed rats (Bianchi et al., 2009). The presence of this tubulin isoform may account for more stable microtubules (Webster and Borisy, 1989), but the function of acetylation still remains unclear. *In vitro* studies assessing the effect of the depolymerizing agent nocodazole, revealed a biphasic effect on α-tubulin isoforms, i.e. a rapid decrease in the tyrosinated α-tubulin followed by a later decrease in acetylated α-tubulin in purified microtubules (Baas et al., 1991). We may hypothesize that the decrease in acetylation might be considered as a delayed marker of microtubular alterations following a prolonged stress exposure. More recently, it has been shown that tubulin acetylation enhanced the recruitment of motor proteins associated to microtubules like kinesin-1 and dynein, leading to an increase of axonal flux and the subsequent release of BDNF (Dompierre et al., 2007). Four-week administration of MAP4343 to stressed tree shrews did not rescue the ratio tyrosinated/detyrosinated α-tubulin, but it prevented the decrease in acetylated α-tubulin. This suggests that MAP4343 may preserve the acetylation of α-tubulin and maintain the axonal function which may be altered in cases of prolonged stress exposure. However, it is certainly premature so far to correlate this specific change of tubulin isoform to any biological function, and therefore to the antidepressant-like efficacy of MAP4343 demonstrated here.

### Conclusions

Taken together, our results demonstrate that MAP4343 exerts a potent and robust antidepressant-like efficacy by preventing deleterious effects triggered by psychosocial stress in tree shrews. The antidepressant-like efficacy of MAP4343 was found similar or better than fluoxetine investigated in the present study (the SSRI failed to prevent cortisol increase and sleep alterations) and other antidepressant drugs previously tested. Importantly, MAP4343 was able to counteract stress-induced behavioral alterations already at the beginning of the treatment suggesting a potential rapid action as observed previously in rats (Bianchi and Baulieu, 2012). The applied duration of the oral administration of MAP4343 (i.e. 4 weeks) corresponds to what is classically set to demonstrate a prolonged efficacy of antidepressant drugs in preclinical studies (Papp et al., 1994) and corresponds to the delay of action of most classical antidepressant drugs in humans.

Our results therefore open new perspectives toward innovative therapeutic strategies in DDs centered on the brain’s microtubular system. Further investigations are in progress to elucidate cellular mechanisms (cerebral plasticity, neurogenesis) underlying such antidepressant-like efficacy.

## Statement of Interest

Etienne-Emile Baulieu is President and one of the founders of MAPREG, a biotech company. Isabelle Villey is the MAPREG CEO and Nicolas Froger is a MAPREG employee. Lucie Paresys and Massimiliano Bianchi have been MAPREG employees at the time of the study. Massimiliano Bianchi has currently equity ownership of Transpharmation Ireland limited. MAPREG hold patents on MAP4343: #WO2004067010 in Europe; #8,034,798 B2 and #12,232, 993 in USA.

## Supplementary Material

Fig. S1, and Table 1, Supplementary Material
