# Supplementary material for: Effects of the Synthetic Neurosteroid: 3β-Methoxypregnenolone (MAP4343) on Behavioral and Physiological Alterations Provoked by Chronic Psychosocial Stress in Tree Shrews
Source: Int J Neuropsychopharmacol. 2015 Oct 17;19(4):pyv119. doi: 10.1093/ijnp/pyv119 (PMC4851265; doi:10.1093/ijnp/pyv119)
Supplement: Fig. S1, and Table 1, Supplementary Material [file Corrected_pyv119_Supplementary_Material_final.docx]

**Supplementary Material**

**SUPPLEMENTARY METHODS**

**Determination of MAP4343 Concentrations in Plasma**

MAP4343 concentrations were measured by Liquid Chromatography tandem Mass Spectrometry (LC MS/MS) following a method developed by Bertin Pharma (Orleans, France). After centrifugation (5 minutes, 4000 rpm at 4°C), 10 µL of internal standard solution (pregnenolone acetate; 1 ng/µL and 700 µL of bidistilled water) was added to plasma (40 µL). After mixing and centrifugation (3 minutes; 4000 rpm at 4°C), samples were prepared using a solid phase extraction (Oasis HLB cartridges,; Waters, Guyancourt, France). Samples were loaded into preconditioned columns: a first wash was performed with bidistilled water and a second with a mix of bidistilled water/acetonitrile (8/2, vol/vol). A final elution with methyl-tert-butyl ether allowed for the collection of products for derivation. This eluted phase was shaken and evaporated under a nitrogen stream at approximately 40°C. The residue was then dissolved in hydroxylamine solution (0.75 mol/L) and heated at 90°C for 30 minutes. Bidistilled water and methyl-tert-butyl ether were added and samples were shaken 5 minutes and centrifuged (4000 rpm for 5 minutes at 4°C). The organic phase was evaporated under a stream of nitrogen at 40°C. The residue was dissolved in a mix of bidistilled water/acetonitrile (15/85, vol/vol) containing 0.1% acetic acid. After centrifugation (4000 rpm for 3 minutes at 4°C), the mixture (5 µL) was injected into an analytical column (XTerra MS C18, 2.1x100 mm, 5 µm; Waters) for liquid chromatography/tandem mass spectrometry analysis.

**Measurements of Urinary Hormones**

Urinary samples were collected each morning under dimmed light before the lights were turned on, as previously described by Schmelting et al. (2014). Aliquots were kept frozen until analyses. Noradrenaline concentrations were quantified by high-performance liquid chromatography (Medizinisches Labor Bremen, Germany) while free cortisol levels were measured by a luminescence immunoassay kit (IBL International, Hamburg, Germany). To compensate for the various physiological urine dilutions, levels of the hormones were normalized and expressed in ng/µmol of creatinine (Roche Modular P800 clinical chemistry analyzer with creatinine reagents according to Jaffe’s method).

In previous studies, we measured urinary adrenaline in stressed tree shrews. The results revealed only a transient increase of the catecholamine in the urine of stressed animals (Fuchs et al., 1993). Therefore, we decided to measure urinary noradrenaline instead of adrenaline, because the first one showed a robust, long-lasting, and significant increase during psychosocial stress; this effect was reproducible over many years (eg, see Kramer et al., 1999; Kozicz et al., 2008; Kohlhause et al., 2011). In studies investigating the effects of different drugs in psychosocially stressed tree shrews, we analyzed only urinary noradrenaline and consequently only noradrenaline was analyzed in the present study too.

**Infrared Western-Blot Detection of Hippocampal Microtubular Proteins**

Western-blot analyses were performed on hippocampus extracts in 2 groups of stressed tree shrews, treated by MAP4343 or untreated, respectively. Data were compared with those obtained in a group of unstressed animals left undisturbed in their cages during the whole experimental period. All tree shrews were sacrificed by decapitation after the 8-week experimental period. The hippocampi were dissected on an ice-chilled plate and immediately frozen on dry ice and stored at -80°C until analysis. Hippocampi were homogenized in lysis buffer (5 mM -Tris HCl, 2 mM -EGTA, 2% Protein Inhibitor Cocktail; Sigma-Aldrich, Quentin Fallavier, France) at 4°C through sonication (Branson Sonifer-450). Total protein concentrations were determined using a Bradford colorimetric assay (Sigma-Aldrich). Homogenates were then treated for protein denaturation with Laemmli buffer: 62.5 mM -Tris-HCl (pH 6.8), 20% glycerol, 2% sodium dodecyl sulfate, 5% β-(2)-mercaptoethanol, and 0.006% bromophenol blue. Electrophoreses were performed on 26-well gels containing 10% bisacrylamide/trisacrylamide (NuPAGE, Invitrogen, Cergy Pontoise, France). Proteins were transferred on to PVDF membranes (Millipore) using a dry blot system (Invitrogen). Membranes were blocked for 1 hour at room temperature in a solution of Odyssey Blocking Buffer diluted (1:2) in PBS (OBB/PBS buffer, ScienceTec, Villebon-sur-Yvette, France). They were then incubated with primary antibodies diluted in OBB/PBS + Tween-20, 0.2%: (1) the monoclonal mouse antibodies against total α-tubulin (1:2000; overnight at 4°C; clone DM-1A, Sigma-Aldrich), tyrosinated α-tubulin (1:2000; 1 hour at RT; clone TUB-1A2; Sigma-Aldrich) or acetylated α-tubulin (1:4000; 1 hour at RT; clone 6-11B-1; Sigma-Aldrich), and (2) the polyclonal rabbit antibody against detyrosinated α-tubulin (1:4000; 1 hour at RT; Chemicon, Temecula, CA). After 4 washings (15 minutes in OBB/PBS + Tween 20 0.1%), the membranes were incubated for 1 hour at RT with secondary antibodies: the polyclonal goat anti-mouse IRDye 680 (infrared detection at 680 nm, Li-Cor, Lincoln, NE) or the polyclonal goat anti-rabbit IRDye 800 (infrared detection at 800 nm, Li-Cor), both diluted at 1:10 000 in OBB/PBS + Tween-20, 0.2% + SDS, 0.1%. Following 4 washes (15 miutes, in OBB/PBS + Tween-20, 0.1%), wet membranes were digitalized with the Odyssey scanner to detect the infrared signal of bands. Integrated intensities were then measured using the Odyssey V3.0 software (LI-COR, ScienceTec). This protocol allowed for the simultaneous detection of α-tubulin isoforms on the same gel (Figure 6A), permitting the calculation of the Tyr/Glu-Tub ratio, considered an index of microtubule dynamics ([Bianchi et al., 2006](#_ENREF_6)).

**Statistical Analyses**

All data were obtained from independent animals and expressed as mean ± SEM. Data were collected daily, and weekly means were calculated. For the control and stress periods, only data from the last week (control: Wk 2; stress: Wk4) were presented for clarity. For each group, data were obtained independently from the other group, and values measured during the control week (Wk 2) are considered as the proper control values of those measured during the first stress period (Wk 4) in order to observe the effects of stress. Similarly, the values measured during the first stress period (Wk 4) are considered as the reference values of those measured during the last stress period (Wk 8) in order to observe the effects of treatment. Accordingly, 1-way ANOVA analyses for repeated measures were performed to compare variances within each experimental group (untreated, MAP4343-, or fluoxetine-treated group, respectively) and followed by the Fisher LSD posthoc test to compare the means at each weekly data point. In addition, means of difference scores were compared using a Student’s *t* test in case of comparison of values from 2 experimental groups (behavioral data) or using a 1-way ANOVA followed by the Fisher LSD posthoc test when comparing values from 3 experimental groups.

For Western-blot studies, a nonparametric Kruskal-Wallis ANOVA was performed followed by the Dunn’s posthoc test to compare means between groups.

All tests were performed using InVivoStat Statistical Software (Huntingdon Life Sciences Ltd, Huntingdon, UK). Differences between groups were considered significant at the 95% confidence level (*P* ≤ .05).

**RESULTS**

**Body Weight**

Psychosocial stress in animals receiving vehicle led to a significantly decreased body weight of approximately 5% at the end of experimental period (F_5,35_ = 2.14, *P* ≤ .05; 1-way ANOVA) (supplementary Figure 1A). This decrease was observed during each week of the stress period as compared to the control period (Wk 2). In the MAP4343-treated group, stress also induced a decrease in body weight (F_5,35_ = 11.41, *P* < .001; 1-way ANOVA) (supplementary Figure 1A), and the administration of MAP4343 did not rescue the reduced body weight at any time during the 4 weeks of administration. Finally, in the fluoxetine-treated group, the body weight was found significantly reduced at each week of the stress period (F_5,35_ = 10.34, *P* < .001, 1-way ANOVA) (supplementary Figure 1A), showing that fluoxetine did not rescue the stress-induced decrease of body weight, similarly to MAP4343. Measurements of body weight gain between Wk8 and Wk4 revealed no significant difference between the 3 groups (F_2,21_ = 0.89, *P* = .42, 1-way ANOVA) (supplementary Figure 1B).

**REFERENCES**

Bianchi M, Fone KF, Azmi N, Heidbreder CA, Hagan JJ, Marsden CA (2006) Isolation rearing induces recognition memory deficits accompanied by cytoskeletal alterations in rat hippocampus. Eur J Neurosci 24:2894–2902.

Fuchs E, Jöhren O, Flugge G (1993) Psychosocial conflict in the tree shrew: effects on sympathoadrenal activity and blood pressure. Psychoneuroendocrinology 18:557–568.

Kohlhause S, Hoffmann K, Schlumbohm C, Fuchs E, Flugge G (2011) Nocturnal hyperthermia induced by social stress in male tree shrews: relation to low testosterone and effects of age. Physiol Behav 104:786–795.

Kozicz T, Bordewin LA, Czeh B, Fuchs E, Roubos EW (2008) Chronic psychosocial stress affects corticotropin-releasing factor in the paraventricular nucleus and central extended amygdala as well as urocortin 1 in the non-preganglionic Edinger-Westphal nucleus of the tree shrew. Psychoneuroendocrinology 33:741–754.

Kramer M, Hiemke C, Fuchs E (1999) Chronic psychosocial stress and antidepressant treatment in tree shrews: time-dependent behavioral and endocrine effects. Neurosc Biobehav Rev 23:937–947.

Schmelting B, Corbach-Sohle S, Kohlhause S, Schlumbohm C, Flugge G, Fuchs E (2014) Agomelatine in the tree shrew model of depression: effects on stress-induced nocturnal hyperthermia and hormonal status. Eur Neuropsychopharmacol 24:437–447.


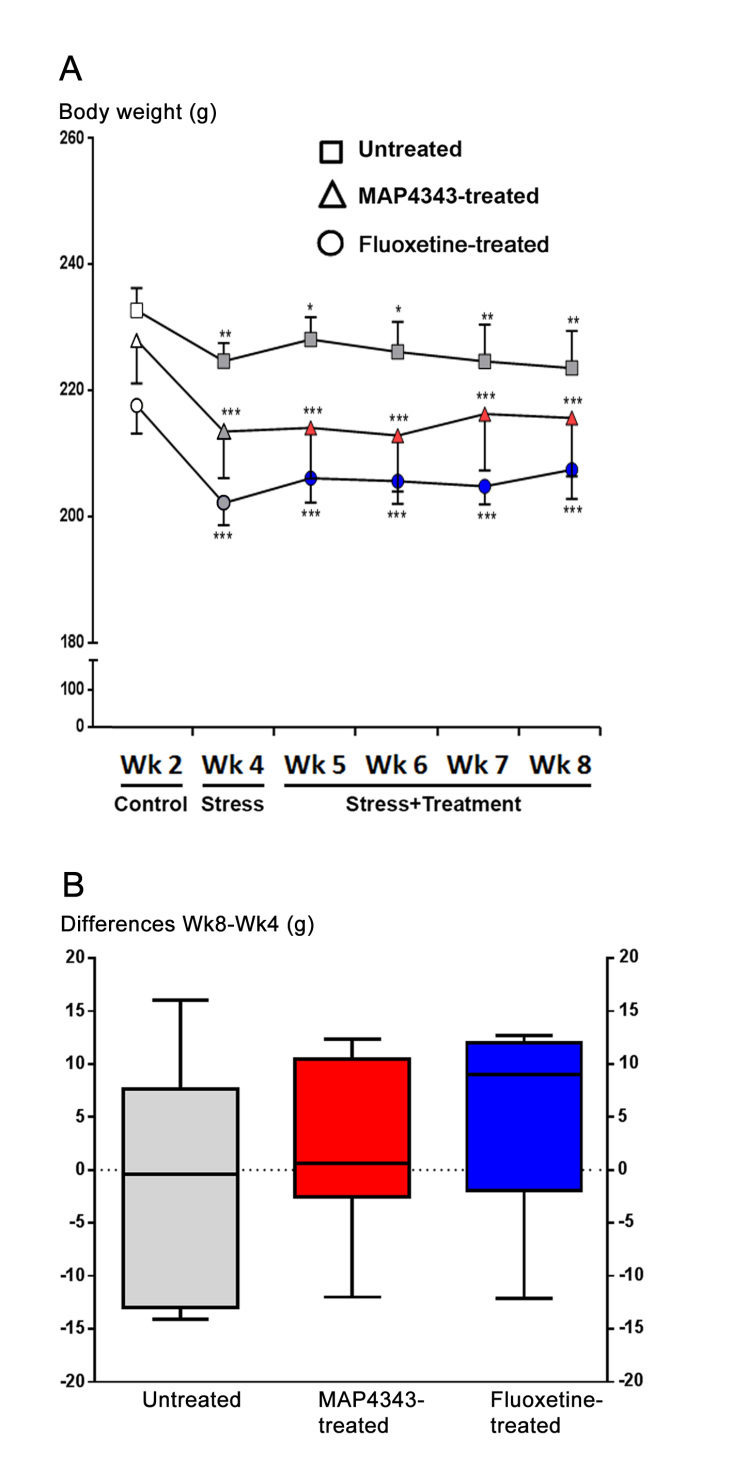


Figure 1. Effects of MAP4343 and fluoxetine on body weight in stressed tree shrews. (A) Curves representing the body weight during the whole experimental period in tree shrews receiving vehicle (squares) or treated with MAP4343 (triangles) or fluoxetine (circles). Data expressed as gram are weekly means (±SEM) calculated from daily measurements in each independent animal (n = 8). White items represent the values obtained during the control period, grey items represent the values obtained during the stress period without treatment, and red or blue items represent the values obtained during the stress periods with MAP4343 or fluoxetine treatment, respectively. **P* < .05, ** *P* < .01, and *** *P* < ..001 compared with control period (Wk 2) (1-way ANOVA for repeated measures followed by a Fisher’s LSD test to compare means from each week within each respective group). (B) Boxplots showing the gain of body weight between the beginning and the end of the stress period in untreated (grey bar), MAP4343-treated (red bar) or fluoxetine-treated (blue bar) group. Data are difference scores (in grams) obtained between Wk8 and Wk4.
